# MRI relaxation parameters predict functional outcome after experimental myocardial infarction

**DOI:** 10.1186/1532-429X-18-S1-O57

**Published:** 2016-01-27

**Authors:** Sebastian M Haberkorn, Christoph Jacoby, Ulrich Flögel

**Affiliations:** 1 grid.14778.3d0000000089227789Department of Cardiology, Pneumology and Angiology, University Hospital Duesseldorf, Duesseldorf, Germany; 2 grid.411327.20000000121769917Molecular Cardiology, University of Duesseldorf, Duesseldorf, Germany

## Background

Characterization of infarcted myocardial tissue by current cardiovascular magnetic resonance (CMR) methods is predominantly carried out after i.v. application of Gadolinium (Gd)-based contrast agents (CA). However, recent advances in CMR enable the acquisition of parametric maps making use of endogenous tissue properties for diagnostic purposes, which may supersede the need for injection of CA. In the present study, we systematically compared myocardial tissue characterization by Gd-based techniques with intrinsic T1/T2 mapping and their correlation with local cardiac function after experimental myocardial infarction (MI).

## Methods

**Figure 2:**
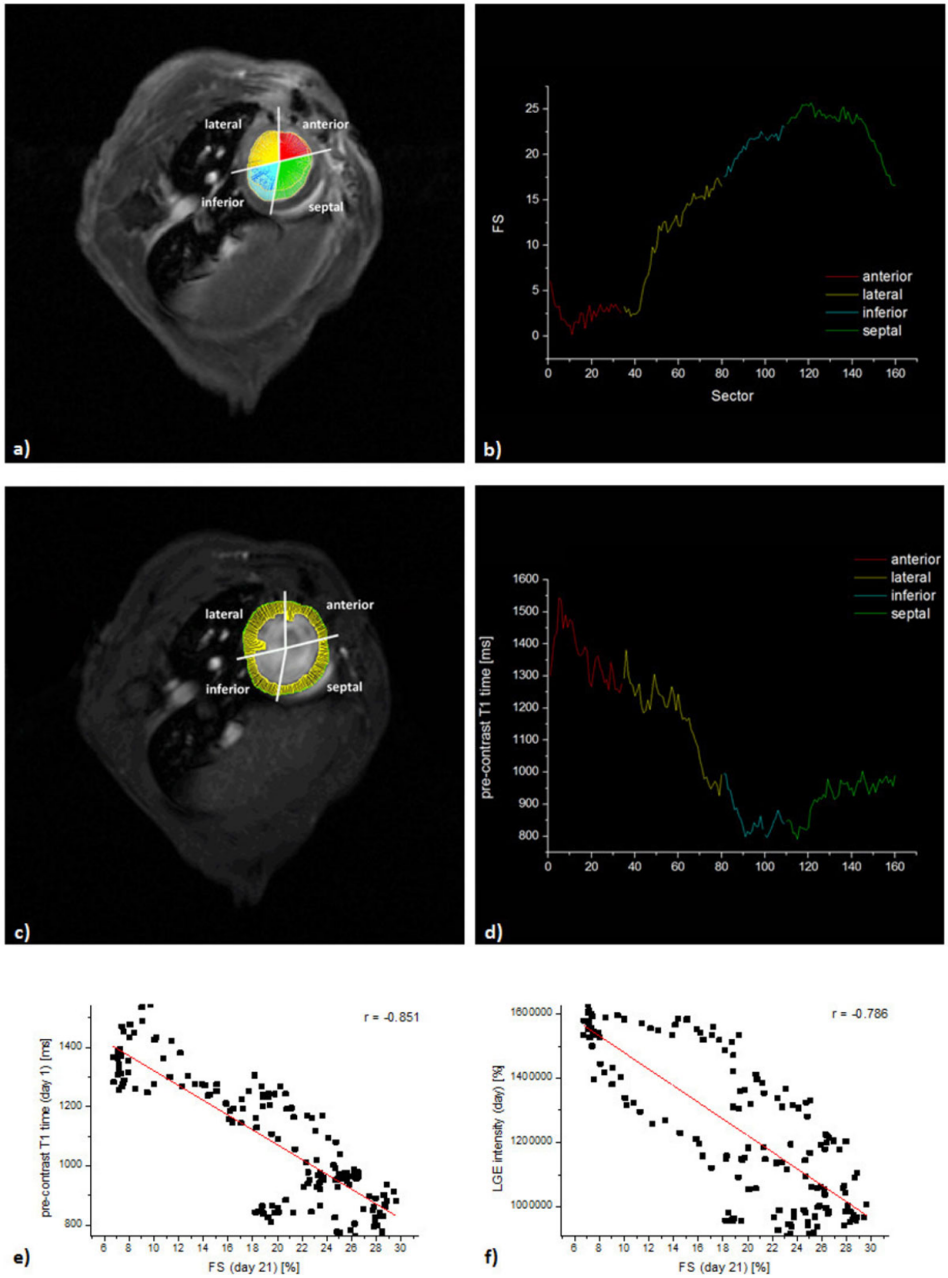
**Regional wall movement and parametric analysis over 160 sectors**. **a)** Local wall movement from end-diastole to - systole with color-encoding for the anterior, lateral, inferior, and septal sections of the LV. **b)** Sectorial analysis of wall movement (give as FS [%]) with poor contractile function particularly in the anterior wall. **c)** ROIs visualizing endo- and epicardial borders of the LV in end-diastole for pre-contract T1 calculation in each sector. **d)** regional pre-contrast T1 analysis clearly reveals enhanced T1 values in sectors corresponding to impaired local function in (b). **e)** Correlation and linear fit of pre-contrast T1 from day 1 with local function (FS) at day 21 (r = -0.851, p < 0.0001). **f)** Correlation and linear fit of LGE from day 1 with local function (FS) at day 21 (r = -0.851, p < 0.0001).

To this end, we used a murine model of MI and monitored the mice over a period of 21 days (n = 9). MI was induced by chronic ligation of the distal left anterior descending artery (LAD) and comprehensive CMR was performed at 9.4T including cine movies, pre- and post-contrast T1, T2 mapping as well as LGE (Fig. 1). To overcome heart rate associated problems during T1 mapping we used a retrospectively triggered fast low-angle shot sequence with variable flip angle analysis providing constant repetition time and maintaining steady-state conditions. Using this approach high-quality T1 maps could be generated in line with literature data (pre- 1001 ± 8.6 ms and post-contrast 289.3 ± 5.9 ms). Extracellular volume (ECV) was calculated from T1 of myocardium and blood pool pre- and post-contrast administration (32.2 ± 2.6%). T2 maps were generated from a gated multi-echo spin-echo sequence (18.8 ± 2.4 ms). For regional correlation of parametric maps and fractional shortening (FS), all data sets were analyzed over 160 sectors covering the entire left ventricle (LV) (Fig. 2).

**Figure:**
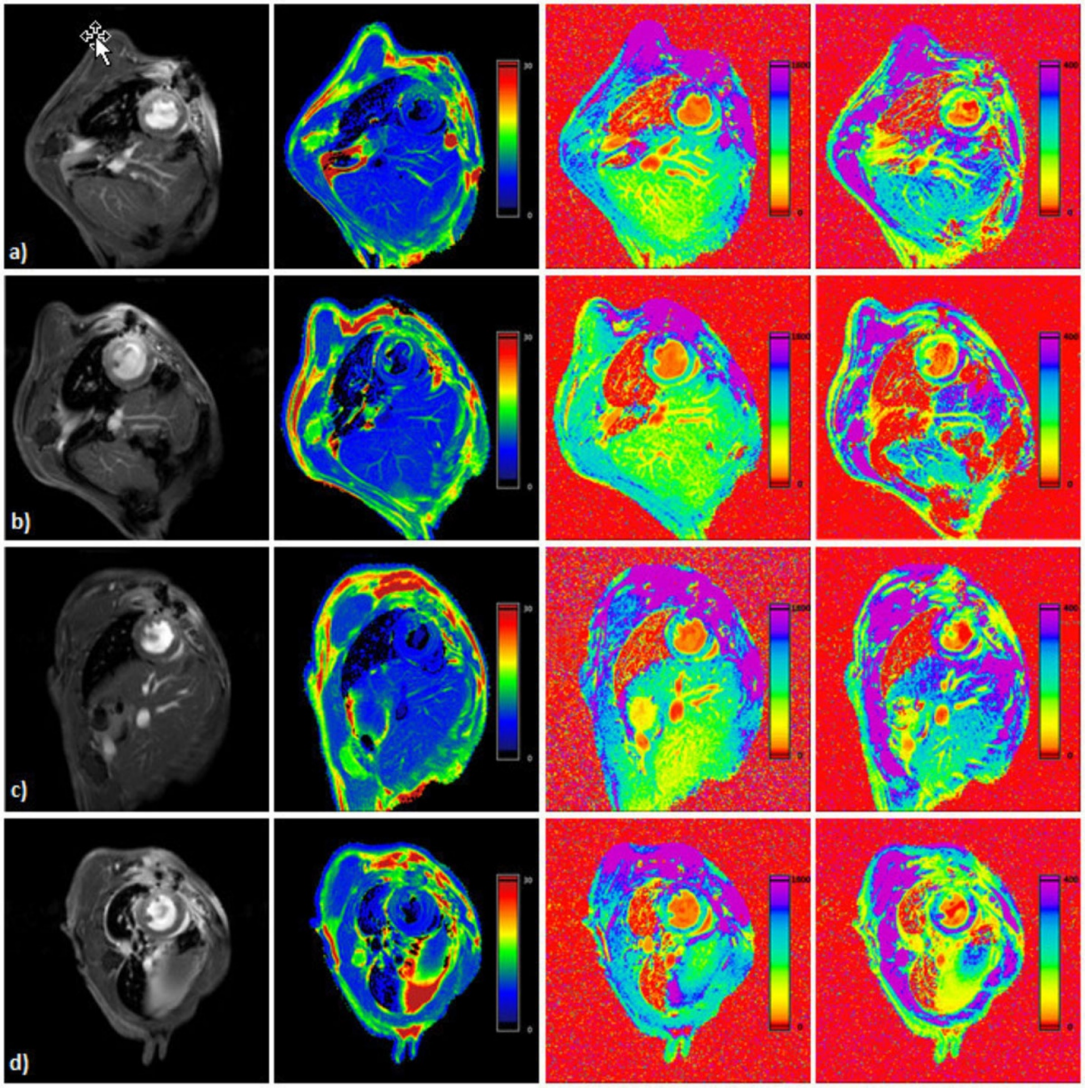
Figure 1

## Results

Longitudinal analysis of mice after MI revealed substantial alterations in MR parameters: At day 1 after MI pre-contrast T1 and T2 increased up to 1461 ± 19.2 ms and 37 ± 0.5 ms, respectively, while post-contrast T1 dropped down to 173.9 ± 5.7 ms in infarcted myocardium (Fig. 1). Concomitantly, ECV increased to 48.6 ± 1.9%. To evaluate the prognostic power of the individual measures for functional outcome, data acquired at day 1 were correlated to local wall movement determined at day 21 (Fig. 2). Quantitative analysis of 160 sectors covering the entire LV revealed for all parameters significant agreement with the later outcome (pre-contrast T1 r = -0.851; T2 r = -0.700; post-contrast T1 r = 0.606; ECV r = -0.691; LGE r = -0.786, all p < 0.0001). Surprisingly, pre-contrast T1 maps on day 1 showed an even better correlation with the FS 21 days after MI than LGE (-0.851 vs. -0.786).

## Conclusions

The present study shows that pre-contrast T1 mapping with variable flip angle analysis carried out 1 day after MI predicts the functional outcome after 21 days at least as reliable as LGE. Compared to the latter technique providing plain signal enhancement, the current approach determines quantitative maps with a large dynamic range, which may pave the way for reliable myocardial tissue characterization without any CA.

